# Concept elicitation and development of a patient-centric conceptual disease model in chronic pruritus of unknown origin

**DOI:** 10.1186/s41687-026-01092-3

**Published:** 2026-06-11

**Authors:** Michael Acquadro, Efstathios Zikos, Carla Dias Barbosa, Karen Bailey, Ariane Dubost-Brama, Eilish McCann, Ashish Bansal, Donia Bahloul, Ella Brookes

**Affiliations:** 1 Patient Centered Research, Evidera, Hammersmith, UK; 2 Sanofi, Gentilly, France; 3 Regeneron Pharmaceuticals Inc., Tarrytown, NY USA; 4 Sanofi, 410 Thames Valley Park Dr, Earley, Reading, Berkshire RG6 1PT UK

**Keywords:** Concept elicitation, Conceptual disease model, Chronic pruritus of unknown origin, Symptom, Impact, Patient perspective, Patient-centric model, Qualitative research

## Abstract

**Background:**

In some cases of chronic pruritus, no identifiable primary cause can be found, whether dermatologic, systemic, neurologic, or psychogenic. In such cases, the disease is referred to as CPUO. To date, little qualitative research has been conducted to understand patient perspectives and a patient-centered conceptual disease model (CDM) for CPUO is lacking. The aims of this study were: to explore the signs, symptoms, and impacts that patients associate with CPUO; and to use these qualitative findings to develop a conceptual disease model (CDM).

**Methodology:**

Telephone or web-based semi-structured qualitative interviews were conducted with American adults ≥ 18 years old with a clinician diagnosis of CPUO. Participants were identified at five US clinical sites and interviewed between November 2022 and September 2023. Their interview transcripts were coded using ATLAS.ti, and analyzed to determine the main signs, symptoms, and quality of life impacts of CPUO. Health status over the past month was self-rated.

**Results:**

The 20 participants were predominantly female (70%), and White (50%) or Asian (40%). Their mean (SD) age was 42.7 (22.4) years. Participants were generally single (65%), employed (65%), and in good (70%) to very good health (15%). All participants reported itch. Other common symptoms included raw skin (85%), stinging (70%), burning (70%), and dry or rough skin (70%). Participants declared itch (55%) to be the worst symptom. In terms of domains impacted, participants with CPUO frequently cited day-to-day activities (90%), sleep (80%), and work or school (70%). Participants reported the domain on which CPUO had the worst impact was sleep (59%). A patient-centric CDM was developed based on these findings.

**Conclusions:**

This first non-interventional qualitative study of adults with CPUO has identified, described, and substantiated the symptoms and impacts of CPUO, which will help define key concepts for patients with CPUO. Itch was the disease-defining symptom of CPUO, and sleep disturbance was its main impact. These and other findings could help physicians make informed clinical decisions about CPUO, and help researchers design CPUO studies with appropriate endpoints.

**Supplementary Information:**

The online version contains supplementary material available at 10.1186/s41687-026-01092-3.

## Introduction

Chronic pruritus (CP) is defined as itch that lasts for six weeks or longer, and it affects approximately one in five individuals at some point in their lifetime [1]. Persistent itch can significantly impact various aspects of a person’s life, including sleep, mood, and overall quality of life [2]. In 8–15% of patients with CP, no identifiable cause can be found, whether dermatologic, systemic, neurologic, or psychogenic. Itch in this subset of patients is referred to as chronic pruritus of unknown origin (CPUO) [3].

There is a lack of research specifically exploring how patients with this condition experience CPUO and describe its signs, symptoms, and impacts. Understanding these patient perspectives could provide valuable insights for consideration in future drug development and treatment strategies [4]. Qualitative research is valuable in supporting drug development programs, especially on the selection of relevant patient-reported outcomes (PRO) [5]. A study was therefore conducted with the objective of identifying, describing, and substantiating concepts that are important and relevant to patients with CPUO. Since there is no existing conceptual disease model (CDM) describing CPUO, the aim was to develop a CDM that captures key CPUO aspects as experienced by patients. This CDM could serve as a foundation for guiding future research and developing effective CPUO treatments.

Methods.

### Study design and participant identification

This non-interventional, qualitative study involved concept elicitation interviews in US adults with CPUO. Medical staff at five US clinical sites employed a purposive sampling method to identify participants via medical records and clinical databases. These clinical sites screened each potentially eligible participant either during a routine clinical visit or over the phone using an institutional review board (IRB)-approved screening script. Screening and eligibility were documented on an IRB-approved eligibility and recruitment form. The study protocol, informed consent forms, and participant-facing materials were approved by the IRB Advarra (Columbia, MD) and adhered to current Health Insurance Portability and Accountability Act (HIPAA) regulations. This qualitative research was conducted in accordance with the patient-focused drug development (PFDD) guidelines [5, 6].

### Participant eligibility criteria

Clinicians confirmed that participants met eligibility criteria and had a clinician-confirmed diagnosis of CPUO. Eligible participants were ≥ 18 years old and had CP for ≥ 6 months that affected at least two bodily areas (e.g., legs, arms, and trunk). The origin of CP was also unknown at the time of screening.

Other primary causes of CP (e.g., dermatological conditions, systemic conditions, neuropathic origin, psychogenic origin, and drug-related) were specifically excluded. Participants also had a history of inadequately controlled CP and recent moderate-to-severe itch. Itch severity was also reported by participants using a PRO measure, the Patient Global Impression of Severity [PGIS] of Pruritus. PGIS is a one-item categorical scale for patients to assess pruritus severity over the past week as “none”, “mild”, “moderate”, or “severe”. Following screening, clinicians obtained written consent, along with permission for the participant’s contact details to be passed to the interviewers.

### Interviewers

Experienced qualitative researchers were selected as interviewers and trained on the disease area. A pilot interview was conducted by each interviewer and assessed to determine whether revisions to the interview guide were required. In addition, during the first interviews, a senior qualitative researcher evaluated each interviewer’s communication and interview techniques and provided feedback. The same researcher reviewed the next interview, to ensure suggestions for improvement had been implemented.

### Concept elicitation interviews

Eligible participants were contacted and enrolled for a single, one-to-one, 90-minute telephone or web-based interview that took place between November 2022 and September 2023. The semi-structured interviews were conducted using an interview guide, which was developed based on findings from a targeted literature review. Details regarding the targeted literature review can be found in Appendix A, including its objectives, search strategy (Table S1), screening process (Figure S1), and data extraction. The semi-structured interview guide is included in Appendix B.

After introducing the study, the interviewers obtained verbal consent to confirm that participants understood the objectives and interview process, and that they were still willing to participate. The interview consisted of five sections—one section on concept elicitation in CPUO (signs and symptoms, impact); and four sections on cognitive debriefing with four itch-related PRO measures, which will be evaluated in a separate analysis (not reported here).

Interviewers first asked participants to describe CPUO-related signs and symptoms they had experienced. The signs and symptoms reported, as well as whether they were reported spontaneously or after prompting, were recorded. Participants were also asked which symptom was the worst and why (Appendix C). Interviewers then asked participants about the impact of these symptoms on aspects of their daily lives, and which impact was the worst and why (Appendix D). Participants next completed a sociodemographic questionnaire (Appendix E). Upon completion of the interview, participants were compensated a nominal amount for their time.

### Analysis and reporting

Interviews were audio-recorded with participants’ consent, and transcribed verbatim. De-identified transcripts contained no mention of protected or personal health information. All de-identified transcripts were uploaded, coded, and analyzed following a content analysis approach using ATLAS.ti, v22 [7–9]. A preliminary coding dictionary was developed based on the interview guide. Two coders then independently coded the first transcript, and their coding was compared for consistency using the inter-rater agreement feature in ATLAS.ti. The coding dictionary was revised based on coding of this first transcript. Three coders then coded the remaining transcripts. New codes were added to the coding dictionary based on concepts that were identified from the interviews. Quantitative data about sociodemographic characteristics were collected via an electronic survey and analyzed using SAS statistical software (SAS Institute Inc.; Cary, NC, USA). Categorical data were summarized as number (percentage), and continuous data as mean (standard deviation).

The key concepts identified in the coding were reviewed and organized into a draft CDM, informed by the Wilson and Cleary conceptual model of patient outcomes [10]. This model, which includes components on biological and physiological variables, symptom status, functional status, general health perceptions, and health-related quality of life, is suited to presenting qualitative research concepts. A clinical expert reviewed the relevance and fit of concepts in CPUO, and some CPUO-specific considerations were included in the Wilson and Cleary model.

Interview transcripts were ordered chronologically and divided into four groups of five interview transcripts before being evaluated for concept saturation. Concept saturation for new CPUO concepts—both signs and symptoms, and impact—was assessed sequentially in each group. Concept saturation, the point at which additional data collection and analysis yields no new concepts, was considered as being reached when the number of new concepts was < 5% of the total number of unique concepts reported [11]. In qualitative research, over 90% of symptom concepts typically emerge by the 15th interview [12].

## Results

### Population

Of the 31 eligible participants contacted, 20 participants enrolled. Of the 11 participants who did not enroll, three declined, three were unavailable, and five did not respond after three attempts by an interviewer to schedule an interview. Participants were predominantly female (*n* = 14/20, 70%) and White (*n* = 10/20, 50%) or Asian (*n* = 8/20, 40%; Table 1). Most were non-Hispanic (*n* = 19/20, (95%). Their mean age was 42.7 (22.4) years, with a mean duration of 7.4 (10.2) years since their initial diagnosis. Participants were generally single (*n* = 13/20, 65%), employed (*n* = 13/20, 65%), and in good (*n* = 14/20, 70%) or very good health (*n* = 3/20, 15%). Itch was rated as mild^1^ by eight participants (40%), moderate by 10 participants (50%), and severe by two participants (10%). Data on current treatments were obtained from 19 participants, 14 (74%) of whom were receiving one or more treatments. Current treatments included corticosteroid creams and ointments (58%), antihistamines (16%), and topical calcineurin inhibitors (5%).CPUO Signs and Symptoms

**Table 1:** Participant-reported sociodemographic and clinical characteristics

Characteristic	Total (*N* = 20)
**Age (years)**,** mean (SD)**	42.7 (22.4)
**Female**,** n (%)**	14 (70)
**Ethnicity**,** n (%)**	
Hispanic or Latino	1 (5)
**Racial background**,** n (%)**	
American Indian or Alaska Native	1 (5)
Asian	8 (40)
Black or African American	1 (5)
White	10 (50)
**Current marital status**,** n (%)**	
Single	13 (65)
Married	3 (15)
Divorced	2 (10)
Widowed	1 (5)
Other	1 (5)
**Current living situation**,** n (%)**	
Living alone	5 (25)
Living with a spouse, partner, family, or friends	15 (75)
Employment status*,** n (%)**	
Employed, full time	8 (40)
Employed, part time	5 (25)
Self-employed	1 (5)
Student	4 (20)
Retired	4 (20)
Disabled^†^	1 (5)
Unemployed	1 (5)
**Highest level of education**,** n (%)**	
Secondary/high school	3 (15)
Technical or vocational degree	1 (5)
Some college/university	4 (20)
College/university degree (BA, BSc)	9 (45)
Postgraduate degree (MA, PhD)	3 (15)
**Historical dermatological conditions**,** n (%)**	
Acne	8 (40)
Hives (urticaria)	8 (40)
Other	3 (15)
No other dermatological conditions diagnosed	6 (30)
**Self-rated general health**^**‡**^, **n (%)**	
Very good	3 (15)
Good	14 (70)
Fair	3 (15)
Poor	0
**Patient Global Impression of Severity score**^**§**^, **n (%)**	
None	0
Mild	8 (40)
Moderate	10 (50)
Severe	2 (10)
**Current treatments**,** n (%)**	
No Treatment	5 (26)
Treatment*	14 (74)
Corticosteroid creams and ointments	11 (58)
Antihistamines	3 (16)
Topical calcineurin inhibitors	1 (5)
Other	2 (11)
Missing	1

Abbreviations: BA, Bachelor of Arts; BSc, Bachelor of Science; MA, Master of Arts; PhD, Doctor of Philosophy; SD, standard deviation

* Response options were not mutually exclusive. For employment status, three participants with part-time employment were also students, and the disabled participant was also unemployed due to their disability

† Participant’s disability was unrelated to chronic itch

‡ As rated within the past month

§ Patient-reported outcome measure used for assessing the severity of pruritus

Twenty-one signs and symptoms of CPUO were reported by participants. All 20 participants reported itch—the disease-defining symptom of CPUO. Participants described it as constant, with one participant saying, “*It just never goes away, no matter what I try. No medicines, no anything.*” It was also reported as being “*worst at night*” (Table S2). Other common symptoms experienced by participants included raw skin (*n* = 17/20, 85%), stinging (*n* = 14/20, 70%), burning (*n* = 14/20, 70%), and dry or rough skin (*n* = 14/20, 70%; Table 2 and Figure S2). A participant with raw skin described it as follows: “*If I scratch too much, it could get going down to the raw skin. Very pink*” (Table S2). A participant who reported stinging described it as, “*Stinging, more stinging than burning. And I think the stinging is just from having an open wound.*” Another reported that the burning sensation was related to the condition of their skin: “*When it’s getting to the point of turning into hives, it’ll start to burn*.” Dryness prompted one participant to declare, “*My legs are like an alligator because of the dryness in the skin.*”

**Table 2:** Signs and symptoms of CPUO

Sign/symptom	Patients who experienced a sign or symptom of CPUO, *n* (%)			
	Endorsed the symptom	Endorsed the symptom spontaneously	Endorsed symptom after being prompted	Endorsed as the worst symptom*
Itch	20 (100)	18 (90)	2 (10)	11 (55)
Raw skin	17 (85)	1 (5)	16 (80)	2 (10)
Stinging	14 (70)	0	14 (70)	1 (5)
Burning	14 (70)	0	14 (70)	0
Dry/rough skin	14 (70)	5 (25)	9 (45)	2 (10)
Bleeding/scabbing	13 (65)	4 (20)	9 (45)	1 (5)
Lesion/sores	13 (65)	4 (20)	9 (45)	1 (5)
Tingling	11 (55)	0	11 (55)	1 (5)
Hot/cold sensation	11 (55)	0	11 (55)	1 (5)
Skin pain	11 (55)	0	11 (55)	3 (15)
White or dark crusts on skin	10 (50)	1 (5)	9 (45)	1 (5)
Darkening of or purple skin	10 (50)	2 (10)	8 (40)	1 (5)
Inflamed skin	3 (15)	3 (15)	0	0
Hives	3 (15)	3 (15)	0	1 (5)
Bumps	3 (15)	3 (15)	0	0
Rashes	2 (10)	2 (10)	0	0
Prickly skin	2 (10)	2 (10)	0	0
Flakiness	1 (5)	1 (5)	0	0
Oozing	1 (5)	1 (5)	0	0
Prickling headache	1 (5)	1 (5)	0	0
Swelling	1 (5)	1 (5)	0	0

Abbreviation: CPUO, chronic pruritus of unknown origin

* Some participants chose multiple “worst symptoms”

When asked which sign or symptom was worst, participants most commonly cited itch (*n* = 11/20, 55%), followed by skin pain (*n* = 3/20, 15%), raw skin (*n* = 2/20, 10%), and dry or rough skin (*n* = 2/20, 10%). The other eight signs or symptoms of CPUO described by a single participant as the worst (*n* = 1/20; 5%) are listed in Table 2 and displayed in Figure S3. The reason one participant described itch as the worst symptom was, *“Because it affects my whole life”* (Table S3). A participant who considered skin pain to be the worst symptom reported that it limited their movement: *“It restricts me from moving too much sometimes in those areas*,* and sometimes they cause even more itching.”* The time taken for skin to heal was mentioned by one participant who reported raw skin as the worst symptom: *“I would probably say the raw skin because it takes a while to heal. And when it does heal*,* sometimes it just gets really thick and it’s still itchy. So it feels like it takes forever to heal almost.”* Pain was implicated in the choice of dry or rough skin as the worst symptom by one participant, *“Oh*,* honestly*,* I’d just say the cracking just because of how much it hurts.”*

**Fig. 1:**
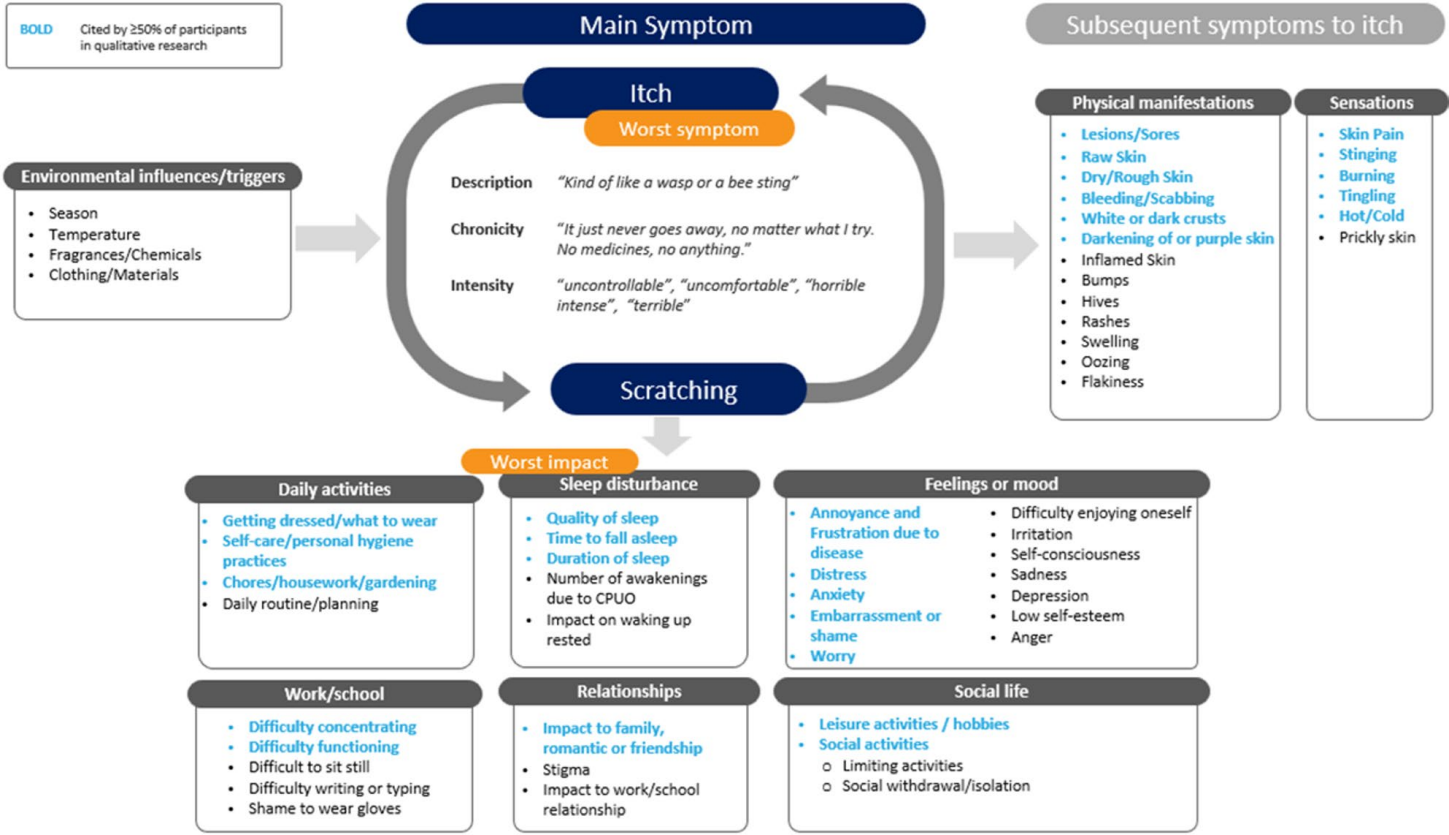
Final conceptual disease model of CPUO. Abbreviation: CPUO, chronic pruritus of unknown origin

Saturation of CPUO sign and symptom concepts was considered achieved, with no new concepts appearing in the last two qualitative interviews of the last of four groups of five transcripts (Table S4).

### CPUO impacts

Thirty-one impacts of CPUO were reported by participants. CPUO commonly disrupted day-to-day activities (*n* = 18/20, 90%), sleep (*n* = 16/20, 80%), work or school (*n* = 14/20, 70%), and feelings or moods (*n* = 13/20, 65%; Table 3). Of those endorsing CPUO impacts on day-to-day activities, more than half (*n* = 11/18, 61%) described difficulties with getting dressed or deciding what to wear. This was often related to the texture of clothing, how it worsened their condition, and how it required them to select clothes carefully. Half of participants (*n* = 9/18, 50%) also reported CPUO impacts on chores and housework, while less than half (*n* = 8/18, 44%) reported impacts on daily routines and planning, due to things taking longer because of symptom irritation or avoidance of certain activities to prevent sweating. According to one participant for whom sweating limited day-to-day activities, *“Yeah*,* I try not to do anything that’s really going to make me sweat. The more I sweat*,* the worse the itch gets. Nothing to do with planning. The daily routine is just avoiding vigorous activities”* (Table S5).

**Table 3:** Impacts of CPUO

Quality-of-life domain	Patients who experienced an impact of CPUO, *n* (%)	
	Endorsed the impact (*N* = 20)	Endorsed as the worst impact (*n* = 17)*
**Day-to-day activities**	**18 (90)**	**2 (12)**
Getting dressed/what to wear	11 (61)	
Self-care/personal hygiene practices	10 (56)	
Chores/housework/gardening	9 (50)	
Daily routine/planning	8 (44)	
**Sleep disturbance**	**16 (80)**	**10 (59)**
Quality of sleep	16 (100)	
Time to fall asleep	15 (94)	
Duration of sleep	11 (69)	
Waking up rested	5 (31)	
Number of awakenings due to CPUO	4 (25)	
**Work or school**	**14 (70)**	**2 (12)**
Difficulty concentrating	11 (79)	
Difficulty functioning	2 (14)	
Difficult to sit still	1 (7)	
Difficulty writing or typing	1 (7)	
Shame to wear gloves	1 (7)	
**Feelings or mood**	**13 (65)**	**3 (18)**
Embarrassment or shame	11 (85)	
Worry	8 (62)	
Annoyance and frustration	7 (54)	
Anxiety	7 (54)	
Distress	7 (54)	
Sadness	6 (46)	
Depression	5 (38)	
Difficulty enjoying oneself	5 (38)	
Low self-esteem	5 (38)	
Irritation	3 (23)	
Anger	2 (15)	
Self-consciousness	2 (15)	
**Social life**	**11 (55)**	
Leisure activities/hobbies	7 (64)	
Social activities	7 (64)	
**Relationships**	**7 (35)**	**1 (6)**
Impact to family, romantic or friendship	7 (100)	
Impact to work/school relationship	2 (29)	
Stigma	2 (29)	

Abbreviation: CPUO, chronic pruritus of unknown origin

* Some participants chose multiple “worst symptoms”

Sixteen participants reported that CPUO impacted their sleep. CPUO also lengthened the time it took for most of these participants (*n* = 15/16, 94%) to fall asleep. More than two thirds (*n* = 11/16, 69%) endorsed how CPUO reduced how long they slept. One participant mentioned, *“I wake up with itching*,* and then I cannot fall asleep again”*. Sleep disturbance was most frequently endorsed as being the worst impact of CPUO (*n* = 10/17, 59%). A participant explained how a lack of sleep could affect his mood: *“I think it would have to be sleep*,* just because when I wake up in the morning*,* not only am I tired*,* which makes me more cranky*,* I also have the itchiness”* (Table S6).

For most participants describing impacts to work and school, these were predominantly related to difficulty concentrating due to itching (*n* = 11/14, 79%). One participant described this as, *“I feel as if I need to take care of it right away. Otherwise*,* I have trouble concentrating.”* CPUO impacts on other work- or-school-related activities—like difficulty functioning (*n* = 2/14, 14%), sitting still (*n* = 1/14, 7%), or writing or typing (*n* = 1/14, 7%)—were less important in comparison to difficulty concentrating. In participants whose feelings or moods were affected by CPUO, most (*n* = 11/13, 85%) reported that CPUO caused feelings of embarrassment or shame. CPUO also often caused worry (*n* = 8/13, 62%), annoyance and frustration (*n* = 7/13, 54%), anxiety (*n* = 7/13, 54%), and distress (*n* = 7/13, 54%).

Saturation of CPUO impact concepts was also considered achieved, with no new concepts appearing in the last three of the 20 interviews (Table S7).

### Model of CPUO

These findings on CPUO signs, symptoms, and impacts were used to develop a patient-centric CDM for CPUO (Fig. 1). Itch is both the defining and the worst symptom of CPUO, and the CDM acknowledges its importance by including the self-perpetuating itch-scratch cycle at its center. Factors that cause, predispose, or exacerbate itch are considered, as well as the outcomes of itch. The former include environmental factors and triggers of itch, season, temperature, chemicals, and clothing. The latter include the main signs and symptoms of CPUO, as well as numerous quality of life domains impacted by CPUO.

## Discussion

To our knowledge, this is the first qualitative study in CPUO. No qualitative studies of CPUO were found by the targeted literature review, only CP studies. These CP studies were nevertheless useful for developing the interview guide, although not specific to our study population. Participants described CPUO in terms of 21 signs and symptoms, and 32 impacts from six quality of life domains. Itch was endorsed as a CPUO symptom by all participants. Participants also frequently endorsed raw skin, stinging, burning, and dry or rough skin as signs and symptoms of CPUO. More than half of participants said the worst symptom of CPUO was itch.

Quality-of-life domains that were impacted by CPUO included day-to-day activities, sleep disturbance, work or school, feelings or moods, social life, and relationships. The most commonly impacted domain of CPUO was sleep disturbance. CPUO decreased the quality of sleep and increased the time taken to fall asleep, in particular. Participants most often endorsed sleep disturbance as the quality-of-life domain worst impacted by CPUO. Other domains commonly impacted by CPUO included day-to-day activities and work or school. CPUO made it especially difficult to get dressed/choose what to wear and to concentrate.

### Comparison with literature from other dermatological conditions

Other qualitative studies have investigated concepts for other skin conditions, e.g., a qualitative study of 21 US adults with prurigo nodularis [13]. Most symptoms (76%) reported by participants with prurigo nodularis were the same as those elicited from participants with CPUO in this study; however, five symptoms (24%)—hives, rashes, prickly skin, flakiness, and prickling headache—were only reported by participants with CPUO and not by participants with prurigo nodularis.

Sleep disturbance has been reported to be a frequent complaint in various chronic pruritic skin conditions [14–17]. An international survey of 30 physicians treating prurigo nodularis confirmed that sleep disturbance was the second most frequent complaint among patients with prurigo nodularis (43%) after itching (100%) [14]. Elsewhere, an analysis of data from the 2013 US National Health and Wellness Survey showed that 349 subjects with atopic dermatitis had a significantly higher rate of sleep disorders than 698 control subjects without atopic dermatitis (33.2% vs. 19.2%; *p* < 0.001). Sleep disturbance associated with other chronic pruritic skin conditions also negatively impacts quality of life [16].

In our study, the impact of CPUO on feelings or moods experienced by most participants was frequently related to embarrassment or shame, which aligns with findings for other chronic pruritic skin conditions in published literature [13, 14, 18]. Psychological distress, such as embarrassment and shame, is also a common complaint of patients with prurigo nodularis [13], and physicians of these patients concur [14]. Other studies have found that prurigo nodularis is strongly associated with depression and anxiety [19]. Elsewhere, people living with a range of itch-related skin conditions have described feeling stigmatized because of scratching and the manifestations of scratching, using terms like “dirty,” “neurotic,” and “contagious” [18].

### Comparison with other CPUO studies

A German retrospective cohort study of 263 patients with CPUO reported findings on itch intensity, itch location, and the burden of CPUO. These German patients were less likely than our participants to be female, (59% versus 70%) and they were older (median age 55 years versus mean age 43 years). The German study found that more than one fifth (22%) of patients had pathological anxiety, and 12% of patients had pathological depression (Hospital Anxiety and Depression Scale score > 11) [20]. In our study, 35% of patients reported being affected by anxiety, and 25% of patients reported being affected by depression. The German study reported that the sleep of 97% of patients was impaired due to pruritus, with a median of 2.0 h of sleep lost per night due to itch [20]. In our study, 80% of patients reported that their sleep was disturbed by CPUO, with 55% reporting that CPUO reduced sleep duration.

### Comparison with other CDMs

CDMs for other skin conditions have been developed and published [18, 21]. A conceptual model of chronic itch of various etiologies was developed by Silverberg and colleagues, based on the five primary components of the Wilson and Cleary model [10, 18]. Components added to the Wilson and Cleary model by Silverberg and colleagues included stigma, social life and relationship problems, emotional disturbances, treatment burden, and treatment response. Although our CDM has many similarities with the CDM for chronic pruritus developed by Silverberg and colleagues [18], there are important differences related to unique CPUO aspects. For example, unlike in the chronic itch model, patients with CPUO experience frustration due to the lack of a definitive etiology, diagnosis, and suitable treatments.

Furthermore, in our CDM, several new concepts related to physical manifestations (e.g., lesions/sores, raw skin, skin pain, and tingling) are identified, and more granular feedback on sleep disturbance has been obtained. One component that is currently lacking in our CDM—compared to the Wilson and Cleary model—is the component on biological and physiological variables [10]. Additional interviews with clinicians would provide insights into the biological and physiological CPUO variables and enhance the CDM.

Such variables could include the type 2 inflammatory pathway, which may play a role in the pathophysiology of CPUO, driving chronic itch [4, 22, 23]. In CPUO, dysregulation of the type 2 inflammatory response can lead to chronic itch, xerosis, and further reductions in the integrity of the skin barrier [4, 22, 23]. Age may also be a factor in CPUO, as the type 2 inflammatory response in skin is exacerbated with age [4, 22, 23].

### Future developments

Findings from this qualitative research could be used to help physicians make informed clinical decisions about CPUO, and help researchers design CPUO studies with appropriate endpoints based on shared decision-making to enhance treatment goals and the holistic management of CPUO. Better awareness of CPUO signs, symptoms, and impacts that patients find important could help physicians better address patient concerns—potentially leading to improved treatment response and increased patient satisfaction.

Developers of future treatments could perhaps target the most common and worst symptoms of CPUO (itch, raw skin, and dry or rough skin). They could also minimize the disruption caused by sleep disturbance. Researchers will have to consider the multi-dimensional nature of impacts (such as sleep disturbance) when measuring them. For example, sleep can be disturbed in a variety of ways (e.g., quality, time to fall asleep, duration, feeling rested, number of awakenings), which has implications for how sleep disturbance is measured [13]. New PRO measures may need to be developed in order to adequately measure outcomes that are important in CPUO but are not currently measured by existing PRO measures for pruritus.

### Limitations

Due to potential selection bias, the key concepts emerging from the interviews may not be generalizable to all patients with CPUO. Additionally, the preferences and experiences of participants who completed the interviews may be systematically different from those who did not wish to participate in the study. For example, our interview sample had low sleep disturbance scores and/or good health in the past week, which leads us to believe that the sample should be considered in the “moderate” range, compared to populations identified in the literature.

Moreover, as the interviews were conducted with participants living in the US, using either online video conferencing or smartphone, with only English-speaking patients, the findings may not generalize to a population that does not have access to electronic devices or non-English-speaking populations in the US. Populations from outside the US, which were not included, may also differ. There may also be different considerations for people of different ethnicities, as skin types may behave differently. For example, African Americans with chronic itch note differences in clinical presentation for select pruritic skin conditions [24, 25], while the skin of Asians (which represented 40% of our sample) may be more sensitive than that of other populations [26]. Consideration should also be given to the fact that age may aggravate CPUO [3].

The inclusion of additional interviews with patients identified by relevant patient organizations could have improved the overall quality of interviews and data analysis. Lastly, although self-reported feelings of anxiety and depression were examined as potential impacts of CPUO, validated questionnaires measuring anxiety and depression were not administered to participants.

## Conclusions

This first non-interventional qualitative study of adults with CPUO has identified, described, and substantiated the symptoms and impacts of CPUO, which will help define key concepts for patients with CPUO. Pruritus concepts underpin the negative impacts on quality of life of CPUO. In terms of impact, sleep disturbance stood out as the worst impact of CPUO. Participant descriptions highlighted the multi-dimensional nature of sleep disturbance and data appear to support the hypothesis that itch affects sleep quality. The development and finalization of a CDM for CPUO will help determine whether existing PROs can be used or adapted for measuring CPUO signs, symptoms, and impacts. As the underlying mechanisms of the disease remain to be confirmed, the proposed CDM could be empirically tested and refined through a quantitative study incorporating multiple PROs (capturing, for example, sleep disturbance, work productivity, and daily functioning). Structural equation modeling could be used to confirm the hypothesized relationships within the CDM. The aim would ultimately be to improve the treatment of patients with CPUO.

## Supplementary Information

Below is the link to the electronic supplementary material.

Supplementary Material 1

Supplementary Material 2

## Data Availability

Additional data that support the findings are available as Supporting Information in the online version of this article.

## Abbreviations

Abbreviation: Meaning
CDM: Conceptual disease model
CP: Chronic pruritus
CPUO: Chronic pruritus of unknown origin
HIPAA: Health Insurance Portability and Accountability Act
PGIS: Patient Global Impression of Severity
PRO: Patient-reported outcome

## Footnotes

1 These patients reported their itch as moderate to severe at screening but reported it as mild during the interview. This may reflect fluctuation of the expression of the disease in those patients.
