# Supplementary material for: Concept elicitation and development of a patient-centric conceptual disease model in chronic pruritus of unknown origin
Source: J Patient Rep Outcomes. 2026 Jun 11;10:151. doi: 10.1186/s41687-026-01092-3 (PMC13486439; doi:10.1186/s41687-026-01092-3)
Supplement: Supplementary file 1 [file 41687_2026_1092_MOESM1_ESM.docx]

**Concept Elicitation and Development of a Patient-centric Conceptual Disease Model in Chronic Pruritus of Unknown Origin**

# Supplementary material

## Table S1. Literature search strategy

| Search # | Strategy | Original* # of hits | Update* # of hits |
| --- | --- | --- | --- |
| 1 | *Disease terms* exp Pruritus [Thesaurus] AND (Idiopathic or unknown origin or unknown caus* or non-lesional skin or without skin lesion).ab,ti.  OR exp Pruritus [Thesaurus] AND (Chronic pruritus not Xerosis not Contact Dermatitis not seborrheic dermatitis not lichen simplex not chronicus not Nummular eczema not stasis Dermatitis not Psoriasis not Scabies not Chronic kidney not Cholestatic liver not Neuropathic).ab,ti.)  OR (Chronic Idiopathic Pruritus or pruritus of unknown origin).ab,ti. |  |  |
| 2 | *Qualitative terms* exp Qualitative Research [Thesaurus] OR exp Focus Groups [Thesaurus] OR exp interview [Thesaurus] OR exp interviews as topic [Thesaurus] OR exp psychological interview [Thesaurus] OR exp Interview, Psychological [Tree] OR exp conceptual model [Thesaurus] OR exp conceptual framework [Thesaurus] OR (qualitative study or Patient journey or patient perception* or disease perception* or patients' perspective or Focus group* or Semi-structured interview or semistructured interview* or structured interview* or in-depth interview* or indepthdescription or face to face interview* or face-to-face interview* or Qualitative interview* or exploratory interview* or individual interview* or Conceptual model* or Conceptual framework or Concept elicitation or Patient* experience* or phenomenological).ab,ti. |  |  |
| 3 | #1 AND #2 | 117 | 66 |

* The original search was conducted on February 25, 2021, and the update was conducted on April 12, 2024.

## Table S2. Participant quotes about the signs and symptoms related to CPUO

| Patient Number | Gender | Age (years) | Illustrative quote about signs and symptoms related to CPUO |
| --- | --- | --- | --- |
| *Itching* | | | |
| P01 | Female | 18 | *Just I feel it's just really, really itchy. I feel it’s kind of possibly eczema, moderate. It gets really itchy at times, on my arm.* |
| P02 | Female | 78 | *Actually, it's all over my body. The itch is worse at night. That's what frustrates the dermatologist, like five different dermatologists. And they don't see much on my skin. But I will break out in a lesion, sometimes really tiny, and sometimes like a three quarters of an inch across. It'll be raised and then eventually, I scratch it, of course, and then it turns up as scar tissue* |
| P03 | Female | 80 | *Well, the main thing is the constant itching. It's usually at night on my legs or in one spot on my neck. If bothers me sometimes during the day, but mostly it's at night. And now I've talked myself into an itching in my back* |
| P04 | Female | 37 | *Just a constant itchy all over my whole body that's like, I mean, I can scratch it and whatever itches at that time will go away but then there's still just more constant itching everywhere. It just never goes away, no matter what I try. No medicines, no anything.* |
| P05 | Female | 24 | *So usually it comes and it goes, but I usually notice like a lot of itching around like in the upper arm. Sometimes going up towards my neck. I feel like it gets a lot worse towards the night or like when I use excessive fragrance and I feel like it gets a lot worse around like allergy season. Sometimes I get it around my legs but it’s very rare, but it’s really like my upper arms that get the most itchy, and then my neck as well.* |
| *Raw skin* | | | |
| P06 | Female | 27 | *And that earlier when sometimes I'm just scratching the same area and then it just becomes like very thin underneath.* |
| P07 | Female | 19 | *I have a terrible itch all over my entire body that's kind of underneath the skin… I can scratch my skin and it's not going to do anything until I find a sharp object and kind of go a little deeper and break some of the skin, to itch it.* |
| P05 | Female | 24 | *Raw skin, partly neck. That’s usually where it’s like the most sensitive, and it does come up there. And that’s before and after, it gets worse just after I touch it.* |
| P08 | Male | 23 | *Sometimes, depending on how much I scratch. I don't know if I’d consider it raw, but more just like the red raw – yeah, I guess you could say like red raw skin.* |
| P01 | Female | 18 | *If I scratch too much, it could get going down to the raw skin. Very pink.* |
| *Stinging* | | | |
| P09 | Male | 69 | *Oh. Yes. Sometimes, kind of like a wasp thing, I'll have that…Kind of like a wasp or a bee sting.* |
| P06 | Female | 27 | *Oh, okay. Stinging, more stinging than burning. And I think the stinging is just from having an open wound and then if it gets touched by something or I like brush up against something, then yeah, it's like stinging sensation, especially like trying to disinfect it or wash it right after.* |
| P05 | Female | 24 | *Stinging it’s not as severe but sometimes I do feel a little bit of stinging.* Interviewer: *Okay, is it caused by anything or is it before or after itching?* P05: *It’s usually a little bit after.* |
| *Burning* | | | |
| P10 | Female | 51 | *Yeah. When it’s getting to the point of turning into hives, it'll start to burn.* |
| P05 | Female | 24 | *Burning, that’s usually after like a constant irritation, and a constant itching. If like you touch it.* |
| P08 | Male | 23 | *Minimally, but a little bit, yeah. More so burning when I try to apply lotion on it.* |
| P03 | Female | 80 | *Usually I get the burning when I scratch so hard that the skin is raw.* |
| P06 | Female | 27 | *It feels like a stinging sensation more than burning. I don't know if how I would differentiate those two.* |
| *Dry/rough skin* | | | |
| P11 | Female | 22 | *I have the itch and it just pops up whenever it's gets really warm or like severe temperatures. And there’s like bumps that form after on my legs and my arms whenever that happens. And sometimes I just scratch without even knowing, in my sleep. It just, it comes and goes and it gets really dry easily in those areas.* |
| P03 | Female | 80 | *Yes. My legs are like an alligator because of the dryness in the skin.* |
| P12 | Male | 22 | *I have itching, irritation, dry skin, and I believe that my skin becomes inflamed, if that's the correct term.* |
| P09 | Male | 69 | *Like I say, it's just an itch and I'll scratch it and I'll make a sore and then it'll have a scab on it and I'll pick that off. Dry skin. And I don't really like to have lotion. I don't like the way it feels.* |
| *Bleeding/scabbing* | | | |
| P13 | Male | 23 | *That’s not as often, just from scratching a lot in my sleep. I can wake up with like some blood. If there was one day where it’s really itchy and I scratch a lot, I’ll bleed as well.* |
| P14 | Male | 75 | *And I scratch it a whole lot and it turns red and if you scratch too much, it’ll bleed.* |
| P08 | Male | 23 | *Symptoms, like it could be symptoms that’s from the itching, so sometimes I can mindlessly itch my skin, and it will bleed or ooze sometimes, just from like constant itching, or sometimes I get like thickened skin, like plaques on where I itch constantly, so just like the secondary symptoms, but other than that, just the constant itching and symptoms coming from the itch.* |
| P11 | Female | 22 | *There's like very little bleeding from like where my nail marks are.* |
| P12 | Male | 22 | *Yes, but it's more of a once in a while. It's not an everyday occurrence.* |
| *Lesions/sores* | | | |
| P02 | Female | 78 | *That's what frustrates the dermatologist, like five different dermatologists. And they don't see much on my skin. But I will break out in a lesion, sometimes really tiny, and sometimes like a three quarters of an inch across. It'll be raised and then eventually, I scratch it, of course, and then it turns up as scar tissue… It's much worse at night and sometimes I wake up with a jolt because the itch is so intense, it wakes me up.* |
| P13 | Male | 23 | *I guess it’s at the place where it itches, sometimes it’s like in the past when I would scratch too much it would cause lesions, like cuts.* |
| P11 | Female | 22 | *They're just like, sometimes the bumps, they're just really, they get thick after I scratch for a really long time. And then sometimes there's like little, if I scratch too much, I notice that there's like fluids that can come out, like very little, but they sometimes come out of those spots.* |
| P06 | Female | 27 | *So like the areas that I'm continuously scratching at, it'll leave behind like that discoloration I described, but it'll be like a focal patch kind of, like localized to that area and sometimes it can bleed.* |
| *Tingling* | | | |
| P13 | Male | 23 | *Well, I guess touching really a sensation of wanting to itch it when I’m trying not to itch it.* |
| P09 | Male | 69 | *Yes, I have peripheral neuropathy that kind of comes and goes, so it's kind of hard to differentiate between them.* |
| P14 | Male | 75 | *If I don't scratch, if I don't rub it or scratch it or put anything on it, yeah, my skin kind of tingles. It feels like it's on the outer layer of the skin tingling.* |
| *Hot/cold sensation* | | | |
| P06 | Female | 27 | *Sometimes if I don't - if I try to hold out and not scratch it, then I'll get like that burning sensation to do it because it feels like hot to touch in that area and then scratching relieves it.* |
| P02 | Female | 78 | *I've had trouble with cold ever since I was probably born. I'm always cold. But that probably doesn't have anything to do with the itch.* |
| P15 | Female | 24 | *I want to say hot sensations, like, it feels like bugs are, like, walking over me…just kind of like throughout my body, and like, it's mainly like my back or like the areas that I've mentioned.* |
| *Skin pain* | | | |
| P05 | Female | 24 | *Skin pain, usually that comes after I start itching it too much.* |
| P12 | Male | 22 | *Yes, that's part of the irritation, the inflammation I was talking about. Interviewer: Okay. I see. With that, just to see if I'm understanding correctly, where it's irritated or inflamed, do you experience pain?* P12: *Yes.* |
| P04 | Female | 37 | *If I don't itch it then it kind of becomes like a, I’m not going to say a burn, but kind of like a burn where it's like really irritating so I have itch it.* |
| P06 | Female | 27 | *I would say it's only painful when it starts to reach like the layers underneath, so when it gets to the point of bleeding or when it's just very raw, I guess that's the way to describe it.* |
| *White or dark crusts* | | | |
| P06 | Female | 27 | *I would say the crust itself is light.* |
| P11 | Female | 22 | *It's like I noticed that once it heals, it does leave like a dark area on my skin.* |
| P16 | Female | 28 | *More so dark. I think after I let, I've let it healed. I do know I get PIH. I'm not sure if that's part of it.* |
| P08 | Male | 23 | *Symptoms, like it could be symptoms that’s from the itching, so sometimes I can mindlessly itch my skin, and it will bleed or ooze sometimes, just from like constant itching, or sometimes I get like thickened skin, like plaques on where I itch constantly, so just like the secondary symptoms, but other than that, just the constant itching and symptoms coming from the itch.* |
| P15 | Female | 24 | *I would say white crust. It almost looks like, I don't even know if it's eczema, but like the crusting, it definitely, like, looks like I have dry skin.* |
| *Darkening/purple skin* | | | |
| P06 | Female | 27 | *It's very itchy, dry, flaky. Sometimes I scratch it and it bleeds, and the skin kind of looks like thick and darker or sometimes redder.* |
| P12 | Male | 22 | *My skin does darken, but I don't think it's ever been purple. There are patches on my skin that are darker than – a darker got a shade then the rest of my skin. And those areas, the itching's a bit more common.* |
| P03 | Female | 80 | *Not normally. Where I scratch and cause it to bleed, it gets a little purplish.* |
| P08 | Male | 23 | *More like darker pigmented skin, but the texture changes a little bit, where the places I itch for long periods of time, like over months or over weeks, especially on my ankles, it will be a little bit darker than the rest of my skin and it feels like thickened or almost calloused from the constant rubbing and itching.* |
| P17 | Female | 66 | *It’s just I have it on the trunk and it’s on the back, and it’s just itching on and off, and then it’s also red, reddish, the area is also reddish.* |
| *Hives* | | | |
| P07 | Female | 19 | Interviewer: *And with that, do you experience other symptoms, either secondary to the itch or just alongside the itch?* P07: *I break out in hives a lot.* |
| P10 | Female | 51 | Interviewer: *So you just mentioned the first symptom, which is hives. You said you get that without medication, when you’re not on medication?* P10: *Yeah. When I'm not on medication, I will get sporadic hives.* |
| *Other symptoms* | | | |
| P12 | Male | 22 | Inflamed skin. *I have itching, irritation, dry skin, and I believe that my skin becomes inflamed, if that's the correct term.* |
| P16 | Female | 28 | Rash. *Pretty much an irritating rash. It's usually very specific. It's only on my upper back. Sometimes when I get like random allergic reactions, like I'm allergic to dogs, I'll start itching at a place where I had contact with that dog. And sometimes sweat makes it worse. And when it's really dry outside, like during the winter and spring seasons, it would also make it act up as well.* |
| P11 | Female | 22 | Bumps. *I have the itch and it just pops up whenever it's gets really warm or like severe temperatures. And there’s like bumps that form after on my legs and my arms whenever that happens. And sometimes I just scratch without even knowing, in my sleep. It just, it comes and goes and it gets really dry easily in those areas.* |
| P10 | Female | 51 | Prickly skin. *It feels like, I don't know, like spiderwebs, or anything that touches your skin is bothersome. Like, it's just like right now I can feel it on my legs a little bit.* |
| P01 | Female | 18 | Flakiness. *Yeah. And also some flakiness of it because of the skin, It's on my arm only. Like where you bend it.* |
| P08 | Male | 23 | Oozing. *Symptoms, like it could be symptoms that’s from the itching, so sometimes I can mindlessly itch my skin, and it will bleed or ooze sometimes, just from like constant itching, or sometimes I get like thickened skin, like plaques on where I itch constantly, so just like the secondary symptoms, but other than that, just the constant itching and symptoms coming from the itch.* |
| P10 | Female | 51 | Prickling headache. *It's like a dull thickness in the brain. In my forehead. Not very often anymore because I am on [medication], which was the first time I got full relief. And I'm on allergy medicine at all, also, which also keeps it at bay. And I take sometimes [medications], which don't seem to help a whole lot. It's more the [medication] than seems to do the job.* |
| P18 | Female | 53 | Swelling. *Practically all night. Because, I mean, at the time, it literally would not stop. The painful, intense itching, and the breaking out, before I was prescribed this special medication, it would literally would not stop. Oh, yes, and something else. I would itch and scratch and my skin would swell up.* |

Abbreviations: CPUO, chronic pruritus of unknown origin; SD NRS, Sleep Disturbance Numeric Rating Scale; WI NRS, Worst Itch Numeric Rating Scale.

* Pruritus severity in the 24 hours before screening, on a scale of 0 (no itch) to 10 (worst imaginable itch).

† Sleep disturbance the night before screening, on a scale of 0 (no sleep loss related to itch) to 10 (I cannot sleep at all due to itch).

## Table S3. Participant quotes about the worst signs and symptoms related to CPUO

| Patient Number | Gender | Age (years) | Illustrative quote about the worst signs and symptoms related to CPUO |
| --- | --- | --- | --- |
| *Itching* | | | |
| P02 | Female | 78 | *Because it affects my whole life. It's difficult to sleep unless I put a lot of a hydrocortisone in on the skin and that's over the counter and that's really not good for your skin in the long run. So I worry about side effects.* |
| P12 | Male | 22 | *The itching. It's the most persistent. The itching is one of the most regular ones, I mean to say. I get itchy on a near daily basis. I have to constantly lotion and apply products that my doctor gives me.* |
| *Raw skin* | | | |
| P06 | Female | 27 | *I would probably say the raw skin because it takes a while to heal. And when it does heal, sometimes it just gets really thick and it's still itchy. So it feels like it takes forever to heal almost. And it's quite bothersome, especially if you, like sometimes when I wash my hands with soap, it’ll sting or if I brush it against something, then it's going to feel like worse than it would be on like skin that doesn't have any issues.* |
| P05 | Female | 24 | *Usually it’s – like when I scratch it, it gets raw. It’s just the pain that I feel afterwards, it’s like – it’s not rashy but it’s like – it just feels like the skin is really dried and then anything it will touch, it just hurts after that.* |
| *Stinging* | | | |
| P04 | Female | 37 | *If I don't scratch it, it turns into… I don’t know what to call it really. Just like I have to. It’s really irritating, like I have to scratch it, or it just keeps bugging me. It won't go away on its own like a normal itch would.* |
| *Dry/rough skin* | | | |
| P13 | Male | 23 | *Oh honestly I’d just say the cracking just because of how much it hurts. Like I mentioned when I put lotion on an area when some spots are cracked, it’ll hurt a lot* |
| *Bleeding/scabbing* | | | |
| P08 | Male | 23 | *Probably the thickened skin and the bleeding, because those are the ones that change the way my skin looks and the way it feels. If it was a constant itching with no visual symptoms, I wouldn’t have minded it as much.* |
| *Lesions/sores* | | | |
| P09 | Male | 69 | *Because I get blood on different things that I don't want to, like my shirts and stuff. Like a shirt that I'm wearing, I'll get blood on it.* |
| *Tingling* | | | |
| P15 | Female | 24 | *Like, it's a very uncomfortable sensation. It's like, I feel like the best way to compare it would be like if you had like a million ants walking over you, and you just can't stop it, and so, like that constant itch is what, like, bothers, and then when you form bumps after you scratch, it like almost makes it worse* |
| *Hot/cold sensation* | | | |
| P07 | Female | 19 | *The hot sensations, because it does get very hot and it's almost intolerable.* |
| *Skin pain* | | | |
| P11 | Female | 22 | *It restricts me from moving too much sometimes in those areas and sometimes they cause even more itching. But then I tend to, if I don't put on any creams or anything, I tend to scratch a little bit more. And I guess it's like an ongoing cycle.* |
| *White or dark crusts* | | | |
| P08 | Male | 23 | *Probably the thickened skin and the bleeding, because those are the ones that change the way my skin looks and the way it feels. If it was a constant itching with no visual symptoms, I wouldn’t have minded it as much.* |
| *Darkening/purple skin* | | | |
| P16 | Female | 28 | *I guess since I'm still, what's the word? Oh, it would make me insecure a little bit, so I would try to wear clothing that would hide my back, just because I know it bothers me.* |
| *Hives* | | | |
| P19 | Male | 60 | *Hives would be the worst. I just itch. It's very irritable. Yeah. Irritable. Yeah. Hives are like that.* |

Abbreviations: CPUO, chronic pruritus of unknown origin; SD NRS, Sleep Disturbance Numeric Rating Scale; WI NRS, Worst Itch Numeric Rating Scale.

* Pruritus severity in the 24 hours before screening, on a scale of 0 (no itch) to 10 (worst imaginable itch).

† Sleep disturbance the night before screening, on a scale of 0 (no sleep loss related to itch) to 10 (I cannot sleep at all due to itch).

## Table S4. Saturation table for signs and symptoms of CPUO

| Sign/symptom | Number endorsing symptom (N=20) | Group 1* (n=5) | Group 2* (n=5) | Group 3* (n=5) | Group 4* (n=5) |
| --- | --- | --- | --- | --- | --- |
| Itch | 20 | X |  |  |  |
| Raw skin | 17 | X |  |  |  |
| Stinging | 14 | X |  |  |  |
| Burning | 14 | X |  |  |  |
| Dry/rough skin | 14 | X |  |  |  |
| Bleeding/scabbing | 13 | X |  |  |  |
| Lesion/sores | 13 | X |  |  |  |
| Tingling | 11 | X |  |  |  |
| Hot/cold sensation | 11 | X |  |  |  |
| Skin pain | 11 | X |  |  |  |
| White or dark crusts on skin | 10 | X |  |  |  |
| Darkening of or purple skin | 10 | X |  |  |  |
| Inflamed skin | 3 | X |  |  |  |
| Hives | 3 | X |  |  |  |
| Bumps | 3 |  | X |  |  |
| Rashes | 2 | X |  |  |  |
| Prickly skin | 2 |  |  | X |  |
| Flakiness | 1 | X |  |  |  |
| Oozing | 1 |  |  | X |  |
| Prickling headache | 1 |  |  |  | X |
| Swelling | 1 |  |  |  | X |
| *Number of new symptoms* |  | *16* | *1* | *2* | *2* |

Abbreviation: CPUO, chronic pruritus of unknown origin.

* X indicates that the concept was reported by at least one subject.

## Table S5. Participant quotes about impacts related to CPUO

| Patient Number | Gender | Age (years) | Illustrative quote about impacts related to CPUO |
| --- | --- | --- | --- |
| *Day-to-day activities* | | | |
| P15 | Female | 24 | *I've actually never thought about that, but yes, I think certain fabrics make it a little bit, like, more noticeable, maybe even worse. I don't know.* Interviewer: *And do you just sort of pick those that don't worsen it and avoid those that do?* P15: *I kind of just scratch. I don't really change out of my clothes.* |
| P05 | Female | 24 | *No, I mean I try not to do the dishes with soap. I try and wear gloves all the time if I do try and do anything like that. I don’t really try and let any chemicals or anything like that touch my skin. But I just- I’m always really mindful to wear the gloves every time I do chores or anything like that.* |
| P07 | Female | 19 | *Yeah I try not to do anything that's really going to make me sweat. The more I sweat, the worse the itch gets. Nothing to do with planning. The daily routine is just avoiding vigorous activities.* |
| P01 | Female | 18 | *Yeah, it would just take me a bit longer because sometimes I'll just get distracted or try to calm myself down so I don't start scratching myself. Or if I'm folding clothes and I'm just sitting there, I'll take frequent breaks to just recollect myself.* Interviewer: *How about your daily routine or planning?* P01: *Yeah, just the same kind of thing where just everything takes longer than other people I would say, just trying to get ready. So I kind of have to plan, account for that time and plan ahead. It's not just 30 minutes. To me it's an hour or even longer.* |
| *Work or school* | | | |
| P12 | Male | 22 | *It’s distracting during work and when I'm not at work, it's still very irritating to me. I feel as if I need to take care of it right away, otherwise, I have trouble concentrating. I remember that when I was at work, I suddenly was very itchy and irritated my skin. I had to basically leave right away so I could take care of it. I always carry lotion and certain skincare products with me because I never know when I actually need to take care of this itching. I haven't lost hours per se, but I've lost at least maybe 10 to 20 minutes per episode because I need to go and take care of the itching before I can actually focus again.* |
| P01 | Female | 18 | *And going out just to school in general, it's just like I don't want to just be in class and scratching myself the whole time. And it just kind of makes it hard to focus when all you're thinking about is the itch on your arms that you have, rather than what's actually going on.* |
| P14 | Male | 75 | *I don't know, if I'm in a meeting and having to just sit still for a long time, sometimes I get to squirming and all, can't do that. I get wiggly and trying to rub my back when I'm sitting in and all that kind of stuff.* |
| P08 | Male | 23 | *When the itching gets really bad and it becomes like sore, sometimes it’s hard to write, because one of my plaques is on my wrist, so it can impact maybe like eating or writing, if I have to write, or typing on the computer…Because I do have to type a lot and I have to write a lot, so it impacts it that way, and also I do work with coworkers, and we’re a very close-knit team with my coworkers and I, so sometimes I have to go to extra measures, so I put on bandages, or make sure that I’m not actively bleeding or weeping, that my scabs don't weep in front of my coworkers.* |
| *Feelings or mood* | | | |
| P01 | Female | 18 | *To my mood especially. Just because, again, like I said, it's an urge. I feel really moody and cranky because I have the skin condition and I can't really -I'm trying to help it and get better, have it get better, but it's obviously hard because it doesn't go away like that. But it does affect my mood because I just it hurts, and I just want to scratch it, but again, I can't. It’s just this resisting temptation. You always, you want to do something but you can't.* |
| P04 | Female | 37 | *Sometimes, I would say, yeah, because it put me in like a depression and stopped me from - after I did everything I could possibly think of to stop and it still never stopped, I really got depressed. Like I can’t figure this out and it’s still not going away.* |
| P10 | Female | 51 | *Oh, it's terrible. I mean, it's a detriment in my feeling of self-esteem and confidence, and it makes me feel dirty and yucky. It's huge.* |
| P12 | Male | 22 | *It does make me more self-conscious. In terms of mood, I might say it makes me a little sad, but I don't have an actual gauge on emotional swings…Anxiety, yes. I don't know if I go as far as depression though… I actually do worry about my skin sometimes.* |
| P06 | Female | 27 | *And then I would probably say it makes me a little insecure about what I wear because obviously, I don't want it to be exposed, in terms of I don’t want anything to touch it and I also don't want people to really see it.* |
| *Social life* | | | |
| P06 | Female | 27 | *Mostly just like swimming in a very chlorinated pool would probably be the only thing that I limit myself on, but I still work out and I still go to the beach and do everything I want to do. I think it's just chlorinated pools is the only thing I really avoid.* |
| P05 | Female | 24 | *Leisure activities or hobbies. Well, I do get like- I get into those moods where it’s really bad that I can’t really work out as much as I’d like. I really like doing hot yoga- I try to do it three or four times a week. When it gets really bad, I try to stay away from that just because I feel like- it doesn’t make it -I just try not to do excessive exercise when that happens.* |
| P04 | Female | 37 | *I stopped going everywhere. Like nobody was allowed to come to my house. I didn't have company. I didn't go anywhere. I didn't hang out with my normal friends, family. I didn't do anything at all, but besides going to work and kids go to school, which is required so I had to.* |
| P07 | Female | 19 | *I cannot go out with friends in the heat or anything, if I'm going to sweat. Going to the gym or anything, I cannot really wear tight clothing and be out with people or anything. And then if I just happen to accidentally forget and scratch for a second, I have to continue scratching.* |
| *Relationships* | | | |
| P08 | Male | 23 | *Well, in terms of family, my parents like to always pester me because they’re not very well-versed in – not in medicine – but like the concept of this itch, so they constantly point it out and tell me like, “Why are you itching? Why are you rubbing?” And I just tell them, “I can’t help it, it’s just something that I just do mindlessly,” so I get berated a lot by my mom specifically about my itching, but I’ve just learned to deal with it, so I guess in terms of family, that's one way, but relationship wise or friend wise, not really, more so my parents.* |
| P12 | Male | 22 | *To my friend relationships. They can get a little awkward because they understand that I have - I haven't told them about this specifically, but I did tell them that I have a little bit of itching…I wouldn't go as far as stigmatized. The situation might seem a little bit awkward, but I think my coworkers and friends mainly understand.* |
| P13 | Male | 23 | *I think it’s when I was younger in school, and not now. But when I was younger I guess I’d get bullied about it so it would kind of affect my mental health as I was growing up. Now it doesn’t bother me no more because I’m older and I was- I lived my life already from when I was younger and growing up. It was like being awake to where I just thought there was something really wrong with me.* |
| *Sleep disturbance* | | | |
| P17 | Female | 66 | *I wake up with itching, and then I cannot fall asleep again…sometimes four times and sometimes nothing, so it varies. I get up and put some alcohol on that area, and then I go back, and then depending, sometimes half an hour or sometimes immediately, so it varies.* |
| P13 | Male | 23 | *Just my lack of sleep causes me to not be as active. And when I’m at work I’m always tired. And yes just not getting enough sleep I would say. I mean I think groggy as well because I’m not getting enough sleep.* |
| P02 | Female | 78 | *I wake up about five to six times at night.* |
| P14 | Male | 75 | *Sometimes it [CPUO symptoms] does because I forget to put cream on it and I realized an hour or so later that's why I'm not going to sleep.* |

Abbreviations: CPUO, chronic pruritus of unknown origin; SD NRS, Sleep Disturbance Numeric Rating Scale; WI NRS, Worst Itch Numeric Rating Scale.

* Pruritus severity in the 24 hours before screening, on a scale of 0 (no itch) to 10 (worst imaginable itch).

† Sleep disturbance the night before screening, on a scale of 0 (no sleep loss related to itch) to 10 (I cannot sleep at all due to itch).

## Table S6. Participant quotes about the worst impacts related to CPUO

| ID | Gender | Age (years) | Illustrative quote about the worst impacts related to CPUO |
| --- | --- | --- | --- |
| *Day-to-day activities* | | | |
| P09 | Male | 69 | Interviewer: *Which one would you say is the worst impact for you?* P09: *The one about having to change my clothes.* Interviewer: *And why is that the worst one?* P09: *Because I usually, like over the weekend I had a brand new shirt that I just first time I wore it and I ruined it because I scratched it, got blood on it. So that's usually the worst for me.* |
| *Work or school* | | | |
| P12 | Male | 22 | *It's because when I'm thinking about some problems or some science equations or situations, experiments, I also have this itching feeling. And every time that if I don't take care of it, it just lingers. It doesn't linger, but it's prominent in my mind. So my attention is divided between my actual work and the itching irritation.* |
| *Relationships* | | | |
| P08 | Male | 23 | *Definitely the social aspect with my family, and the fear of having my coworkers see it, because like I said, if it’s just myself, I don't really mind, because I know I can deal with it, it’s more like when my parents especially point out and constantly ask me, “What are you doing,” this, that, this, that, and then they get upset at me when they don't understand that it’s not that I’m doing it on purpose. That's definitely the worst one, yeah.* |
| *Sleep disturbance* | | | |
| P01 | Female | 18 | *I think it would have to be sleep, just because when I wake up in the morning, not only am I tired, which makes me more cranky, I also have the itchiness. I wake up with the itch. I always have the itchiness every single day, but then when I don't get the right amount of sleep or I'm not well rested, that just adds to the moodiness or crankiness. It just builds upon it. So I would say that. So if I’m more cranky, I wouldn't really want to be that social that day. If I'm tired, doing schoolwork, it kind of has a domino effect on all the other things I mentioned. But with tiredness, so with doing schoolwork, I get more tired easily. I can’t really focus because I'm also tired or just if people ask me to go out to dinner, I'm cranky and I feel if possibly go out, not only am I tired, I'm going to be drained and stuff. So I feel just not being well rested just affects my overall mood as well. And then on top of the itchiness, it doesn't go together.* |

Abbreviations: CPUO, chronic pruritus of unknown origin; SD NRS, Sleep Disturbance Numeric Rating Scale; WI NRS, Worst Itch Numeric Rating Scale.

* Pruritus severity in the 24 hours before screening, on a scale of 0 (no itch) to 10 (worst imaginable itch).

† Sleep disturbance the night before screening, on a scale of 0 (no sleep loss related to itch) to 10 (I cannot sleep at all due to itch).

## Table S7. Saturation table for the impacts of CPUO

| Impact* | Number endorsing impact (N=20) | Group 1 (n=5) | Group 2 (n=5) | Group 3 (n=5) | Group 4 (n=5) |
| --- | --- | --- | --- | --- | --- |
| **Day-to-day activities** | **18** |  |  |  |  |
| Getting dressed/what to wear | 11 | X |  |  |  |
| Self-care/personal hygiene practices | 10 | X |  |  |  |
| Chores/housework/ gardening | 9 | X |  |  |  |
| Daily routine/planning | 8 | X |  |  |  |
| **Work or school** | **14** |  |  |  |  |
| Difficulty concentrating | 11 | X |  |  |  |
| Difficulty functioning | 2 | X |  |  |  |
| Difficulty sitting still | 1 |  |  |  | X |
| Difficulty writing or typing | 1 |  |  | X |  |
| Shame to wear gloves | 1 |  |  | X |  |
| **Feelings or mood** | **13** |  |  |  |  |
| Embarrassment or shame | 11 | X |  |  |  |
| Worry | 8 | X |  |  |  |
| Annoyance and frustration | 7 | X |  |  |  |
| Anxiety | 7 | X |  |  |  |
| Distress | 7 | X |  |  |  |
| Sadness | 6 | X |  |  |  |
| Depression | 5 | X |  |  |  |
| Difficulty enjoying oneself | 5 |  | X |  |  |
| Low self-esteem | 5 |  | X |  |  |
| Irritation | 3 |  |  |  | X |
| Anger | 2 |  | X |  |  |
| Self-consciousness | 2 | X |  |  |  |
| **Social life** | **11** |  |  |  |  |
| Leisure activities/hobbies | 7 | X |  |  |  |
| Social activities | 7 |  | X |  |  |
| **Relationships** | **7** |  |  |  |  |
| Impact to family, romantic or friendship | 7 | X |  |  |  |
| Impact to work/school relationship | 2 | X |  |  |  |
| Stigma | 2 |  | X |  |  |
| **Sleep disturbance** | **16** |  |  |  |  |
| Quality of sleep | 16 | X |  |  |  |
| Time to fall asleep | 15 |  | X |  |  |
| Duration of sleep | 11 | X |  |  |  |
| Waking up rested | 5 | X |  |  |  |
| Number of awakenings due to CPUO | 4 | X |  |  |  |
| *Total of new impacts coded* |  | *22* | *6* | *2* | *2* |

Abbreviation: CPUO, chronic pruritus of unknown origin.

* X indicates that the concept was reported by at least one subject.

## Figure S1. Literature screening process

## Figure S2. Signs and symptoms of CPUO endorsed by participants

Abbreviation: CPUO, chronic pruritus of unknown origin.

## Figure S3. Signs and symptoms of CPUO endorsed as the worst by participants

*Participants were able to select multiple choices for the worst sign or symptom of CPUO

Abbreviation: CPUO, chronic pruritus of unknown origin.

# Appendix A. Targeted literature review

## Methods

### Objectives

The objectives of the targeted literature review were to: identify relevant symptom and impact concepts describing the patients’ perspective of chronic pruritus to develop the interview guide; and inform a conceptual disease model (CDM) representing the experience of suffering from chronic pruritus from the patient perspective.

### Search strategy

A literature review of qualitative studies of chronic pruritus of unknown origin (CPUO) was conducted on February 25, 2020 (Table S1). Using the OVID platform (http://ovidsp.ovid.com/), the Embase, MEDLINE, and PsycInfo databases were used to identify studies on the signs and symptoms of CPUO, or quality of life of patients with CPUO, published between 2013 and 2019. A literature review update performed on April 12, 2024, identified similar studies published between 2019 and 2024.

## Results

The targeted literature review and update identified 183 references, 117 of which were identified by the original literature review and 66 of which were identified by the update (Figure S1). Title/abstract and full-text screening of these references was performed using inclusion criteria on study design, population, and outcomes. Studies included were qualitative studies of patients with chronic pruritus that complied with good qualitative study practice and that described subject interviews or focus groups. Outcomes reported included the patient experience of itch and other associated chronic pruritus symptoms.

Of the 183 references identified, 176 (96.2%) were excluded after title/abstract screening. A further 3 references (1.6%) were excluded after full-text screening. Data were extracted from 4 references (2.2%),^1-4^ including one conference abstract. All references from which data were extracted were identified in the original search. No additional references were found in the literature review update. Data extracted in the literature review included: publication reference; study location; study objective; study population (number, age, and gender); methods (qualitative methodology, data collection mode); concepts of importance to patients; and examples of patient descriptions of chronic pruritus.

## References

1. Pijeaux A et al. Factors that drive disparities in pruritus quality of life among Asian Americans: A qualitative study. J Am Acad Dermatol. 2019;81 (4 Supplement 1):AB64.

2. Suilmann T et al. Usability of validated sleep-assessment questionnaires in patients with chronic pruritus: An interview-based study. Acta Derm Venereol. 2018;98(8):722-7.

3. Weiss M, Weisshaar E. Qualitative interviews on chronic pruritus in haemodialysis patients. Acta Derm Venereol. 2014;94(6):713-4.

4. Bathe A et al. Chronic pruritus - more than a symptom: A qualitative investigation into patients' subjective illness perceptions. J Adv Nursing. 2013;69(2):316-26.

# Appendix B. Semi-structured interview guide

**Participant Identification Number: ________________________________________________________**

**Below is the interview script for the Chronic Pruritus of Unknown Origin interview. It is intended to help focus the discussion but should not be used as a verbatim script; probes and questions may change depending on individual feedback. Additional unscripted probes will be used to gain information or clarification, and may include:**

- Clarification: *I don’t quite understand that.*
- Expressing understanding: *How did you cope/manage with that?*
- Justification: *What makes you say that?*
- Extending narrative: *Tell me a bit more about that.*
- Accuracy: *Let’s see if I’ve got that right.*

**The interview session will be approximately 90 minutes of speaking and writing. The interviewer may adapt the guide to cover the topics in the amount of time allotted for the session.**

*Before starting the interview, the interviewer will share the link to the measures (WI NRS, SD NRS, PGIS, PGIC) with the participant and will ask the participant to complete the measures during the course of the interview. During the interview, the interviewer will confirm that consent has been collected, and verbal consent will also be obtained before starting the interview session. The interviewer will introduce themselves and explain the study background and interview process. A concept elicitation discussion will be conducted first.*

Introduction (5 min)

Background and consent

Good *(morning/afternoon/evening)*. I am [*interviewer name*], a research professional working with Evidera, a group focusing on patient-centered research. Thank you for taking the time to participate in our interview on your Chronic Pruritus of Unknown Origin. You have been recruited to participate in this study because you have Chronic Pruritus of Unknown Origin*.* The purpose of this study is to explore your perceptions of and experiences with your Chronic Pruritus of Unknown Origin and its symptoms and how they impact your everyday life. As part of this interview, we will also be discussing four questionnaires that evaluate your symptoms.

This study will involve one interview session and will take approximately 90 minutes to complete. Please be honest with your answers, and do not be afraid to share your individual opinions, experiences, and perceptions. Remember, there are no right or wrong answers.

The interviews will be audio-recorded so that I can capture everything that you say during the interview and review it later. A word-for-word transcript or written document of the audio-recording will be prepared. Your privacy will be protected, and the transcript will not include any mention of your name or information that could be used to discover your identity. You will be identified only with an identification (ID) number to protect your confidentiality.

Participation in this study is strictly voluntary and you can stop at any time during the discussion without any consequences to your medical care or treatment. You do not have to answer any questions that you do not want to. All information you provide will be used for this research project only. There are no known risks to participating in this study, and there is no direct benefit to you from participation in the study, although information learned from the study may help improve how researchers evaluate and improve treatments for people with Chronic Pruritus of Unknown Origin in the future.

You should have received a link with access to four questionnaires via email. Please open this now. We will be using these later in the interview.

You have electronically signed the consent form confirming that you agree to participate in the study, and as stated on the consent form, today’s interview will be audio-recorded. Is it okay for me to record the conversation today?

**If yes, continue.**

**If no, restate information that is present on the consent form:** As was discussed on the consent form, your name will not be linked with the recording, transcription, or your responses during the interview. Unfortunately, since you do not agree to the recording of this session, you won’t be able to participate in the study. Thank you for your willingness to participate in this study.

Discussion rules

Today’s discussion will focus on your experiences with your Chronic Pruritus of Unknown Origin. There are no right or wrong answers because everyone experiences are different. Please feel free to share your point of view about how Chronic Pruritus of Unknown Origin affects you.

Before we begin, let me suggest a few points that will make our discussion more productive.

- My role here is to ask questions and to listen. I’ll also be summarizing the things you’ve said at times to make sure I understand correctly. I’ll ask questions to obtain your feedback, and I’ll move the discussion from one question to the next, to try to keep us on track so we can finish within the allotted time. Please feel free to ask questions or seek clarification at any time.
- Please speak up. We’re audio-recording the session because we don’t want to miss any of your comments and feedback.
- We’ll be talking together on a first-name basis, but later in the report no names will be included or attached to any of your comments.
- I am not a medical doctor. I am a research professional. My background is in conducting various types of research, so I am not qualified to give out medical advice and I may ask you to clarify anything with which I am not familiar. If you have any questions about your condition or treatment, please contact your medical doctor.
- Do you have any questions before we begin?

**Begin Recorder**: This is participant ID [*insert Participant ID number here*] for study EVM-33630, and today is [*date*]. Do I have your permission to record this interview? And would you please confirm that you have electronically signed the informed consent form?

Part 1: Concept elicitation (10 min)

Experience with chronic pruritus of unknown origin (CPUO)

First, I would like to ask you questions about your current experiences with Chronic Pruritus of Unknown Origin and related symptoms and impacts. How do you like to refer to Chronic Pruritus of Unknown Origin? *If no preference:* I’m going to call it CPUO if that’s alright with you.

1. What kind of symptoms do you experience that are related to CPUO?

[Interviewer: Please enter symptoms the participant indicated in Table 1.]

2. I’m going to read a list of symptoms related to chronic itch that have been brought up by other people with CPUO. Can you tell me if you recognize experiencing any of these?

[Interviewer: Probe for all remaining symptoms in Table 1.]

Table 1. Chronic itch-related symptoms prior to study entry

| Spontaneous or probed? | Symptoms | Notes |
| --- | --- | --- |
| □ Spontaneously mentioned  □ Probed | Itch |  |
| □ Spontaneously mentioned  □ Probed | Lesions/sores |  |
| □ Spontaneously mentioned  □ Probed | Skin pain |  |
| □ Spontaneously mentioned  □ Probed | Burning |  |
| □ Spontaneously mentioned  □ Probed | Stinging |  |
| □ Spontaneously mentioned  □ Probed | Tingling |  |
| □ Spontaneously mentioned  □ Probed | Hot/cold sensation |  |
| □ Spontaneously mentioned  □ Probed | Raw skin |  |
| □ Spontaneously mentioned  □ Probed | Dry/rough skin |  |
| □ Spontaneously mentioned  □ Probed | White or dark crusts on skin |  |
| □ Spontaneously mentioned  □ Probed | Bleeding/scabbing |  |
| □ Spontaneously mentioned  □ Probed | Darkening of or purple skin |  |
| □ Spontaneously mentioned  □ Probed | Other: __________ |  |
| □ Spontaneously mentioned  □ Probed | Other: __________ |  |
| □ Spontaneously mentioned  □ Probed | Other: __________ |  |
| □ Spontaneously mentioned  □ Probed | Other: __________ |  |
| □ Spontaneously mentioned  □ Probed | Other: __________ |  |
| □ Spontaneously mentioned  □ Probed | Other: __________ |  |

Of these CPUO related symptoms you have mentioned [*list all symptoms mentioned*], which would you call the **worst** one? Why?

Impact of CPUO symptoms on daily life

1. How have CPUO symptoms affected your daily life? Can you specifically describe how and which areas of your life are impacted?

[Interviewer: Please enter impacts the participant indicated in Table 2. If not mentioned, probe for the following impacts.]

- Day-to-day activities
- Work or school
- Feelings or mood
- Social life
- Relationships
- Sleep (Also probe: Length of time needed to fall asleep, number of times woken up from CPUO in the middle of the night, duration of awakenings related to CPUO, quality of sleep, duration of sleep, waking up feeling rested)

2. How does your chronic itch specifically impact your life? Probe: What is the most important impact of itch to you?
3. Of all the impacts you have been talking about [*list impacts mentioned*], which would you say is the **worst** one? Why?

Table 2. CPUO impacts prior to study entry

| Spontaneous or Probed? | Impact Description | Notes |
| --- | --- | --- |
| □ Spontaneously mentioned  □ Probed | **Day-to-day activities**  ¨ Chores/housework/gardening  ¨ Daily routine/planning  ¨ Getting dressed/what you wear  ¨ Self-care/personal hygiene practices |  |
| □ Spontaneously mentioned  □ Probed | **Work or school**  ¨ Difficulty concentrating |  |
| □ Spontaneously mentioned  □ Probed | **Feelings or mood**  ¨ Depression  ¨ Anxiety  ¨ Embarrassment or shame  ¨ Sadness  ¨ Worry  ¨ Low self-esteem  ¨ Distress  ¨ Difficulty enjoying oneself  ¨ Other |  |
| □ Spontaneously mentioned  □ Probed | **Social life**  ¨ Social activities (e.g. going out the cinema, bar or dinner)  ¨ Leisure activities/hobbies  ¨ Sports (e.g. swimming) |  |
| □ Spontaneously mentioned  □ Probed | **Relationships**  ¨ Family, romantic or friend relationships  ¨ Work/school relationships  ¨ Stigma |  |
| □ Spontaneously mentioned  □ Probed | **Sleeping**  ¨ Length of time needed to fall asleep  ¨ Number of awakenings related to chronic itch or associated symptoms  ¨ Length of time of awakenings related to chronic itch or associated symptoms  ¨ Quality of sleep  ¨ Duration of sleep  ¨ Waking up rested |  |
| Other: specify | |  |

Part 2: Cognitive debriefing of the WI NRS (20 min)

Thank you. For the next part of the interview, I’d like to focus on the Worst Itch Numeric Rating Scale (WI NRS). Please take a minute to answer the question on your computer.

Cognitive debriefing of the Worst Itch Numeric Rating Scale (WI NRS)

1. What score did you choose?

   On a scale from 0 (no itch) to 10 (worst imaginable itch), how was your worst itch in the past 24 hours? Please select one number

| 0 | 1 | 2 | 3 | 4 | 5 | 6 | 7 | 8 | 9 | 10 |
| --- | --- | --- | --- | --- | --- | --- | --- | --- | --- | --- |

[Interviewer: Say the number participant provided]

2. Can you describe what your itch feels like at that level?

[Interviewer: Ask each question in Table 3. If not spontaneously addressed, ask each probe.]

Table 3. WI NRS debriefing

| Topic | Question | Probes | Notes |
| --- | --- | --- | --- |
| **Understanding** | In your own words, what does this question mean to you? | *What does “no itch” mean to you?*  *What does “worst imaginable itch” mean to you?* |  |
| **Clarity** | Is there anything about this question that is difficult to understand or respond to?  If not, how could it be made clearer? | *What does “worst itch in the past 24 hours” mean to you?*  *Considering you completed the questionnaire in the evening (for example around 6pm) what does “worst itch in the past 24 hours” mean to you?*  *If you have a different level of itch between daytime and night-time how do you determine your itch at the worst moment?* |  |
| **Rewording** | If yes, how would you suggest the question be reworded? |  |  |
| **Recall Period** | What time-period did you think about when you answered the question? | *What does the time frame “in the past 24 hours” mean for you?*  *Considering you completed the questionnaire at in the evening (for example around 6pm) what does “in the past 24 hours” mean to you?* |  |
| **Ease** | How easy did you find it to select a response from 0 to 10? Why? | *How easy or difficult was it to select a response on the scale which accurately represented your worst itch in the past 24 hours?* |  |

Qualitative exploration of meaningful change and thresholds in WI NRS

Thank you. For the next phase of the interview, I would like to explore with you which changes in the WI NRS would matter to you.

Earlier, you told me your itch at its worst was [*share score*] during the previous 24 hours. Next, I’m going to ask you to think how much a change on this 0-10 scale would be important or meaningful to you and why.

[Interviewer: If the participant has difficulties understanding what a “meaningful” change, please explain that is a change that is noticeable and results in a change in their day-to-day life that is important and/ or relevant to them.]

1. From your score [*share score*] during the previous 24 hours, what change would be meaningful or important to you in terms of improvement? In other words, what number on the scale would you move to feel that is a noticeable and meaningful improvement in this symptom?

*Insert Number (severity)*

[Interviewer: Say the number participant provided]

2. Can you describe what your itch feels like at that level? How would your daily life change if you had moved to this level?

**Probes for meaningful improvement**

a. Would an improvement from [*original score reported by the participant*] to [1-point improvement] mean something to you?
i. Probe: What could you do differently? Would this change be meaningful or important to you?
b. Would an improvement from [*original score reported by the participant*] to [2-point improvement] mean something to you?
i. Probe: What could you do differently? Would this change be meaningful or important to you?
c. Would an improvement from [*original score reported by the participant*] to [3-point improvement] mean something to you?
i. Probe: What could you do differently? Would this change be meaningful or important to you?
d. Would an improvement from [*original score reported by the participant*] to [4-point improvement] mean something to you?
i. Probe: What could you do differently? Would this change be meaningful or important to you?

**Meaningful worsening**

1. From your score [*share original score*] during the previous 24 hours what change would be meaningful or important to you in terms of worsening? In other words, what number on the scale would you move to feel that is a noticeable and meaningful worsening in this symptom?

*Insert Number (severity)*

[Interviewer: Say the number participant provided]

2. Can you describe what your itch feels like at that level? How would your daily life change if you had moved to this level?
3. [Interviewer: If not already covered] Is there a time when you were at the worst point 10? Please describe your experience in terms of sleep disturbance (probes: difficulties to fall asleep, number and duration of awakenings, etc.) at the worst point 10.
4. [Interviewer: If not already covered] How about when you were at the middle point? Please describe your experience in terms of sleep disturbance (probes: difficulties to fall asleep, number and duration of awakenings, etc.) at the middle point (5)?
5. [Interviewer: If not already covered] How about if you were at the lower end of the scale (1-2)? Please describe your experience in terms of sleep disturbance (probes: difficulties to fall asleep, number and duration of awakenings, etc.) at the lower end of the scale (1-2)?

I want you to do an exercise with me. If I asked you to give me ranges for worst itch on WI NRS when it’s not present , mild, moderate, and severe…

- What would the not present be? ______ to ______
- What would the mild range be? ______ to ______
- What would the moderate range be? ______ to ______
- What would the severe range be? ______ to ______

Part 3: Cognitive debriefing of SD NRS (20 min)

Cognitive debriefing of the Sleep Disturbance Numeric Rating Scale (SD NRS)

Thank you. For the next part of the interview, I’d like to focus on the **Sleep Disturbance Numeric Rating Scale (SD NRS)**. Please take a minute to answer the question.

1. What score did you choose?

On a scale from 0 (no sleep loss related to itch) to 10 (I cannot sleep at all due to itch), how would

you rate your sleep last night?

*Insert Number (severity)*

[Interviewer: Say the number participant provided]

2. Can you describe what your sleep loss due to itch looks like at that level?

[Interviewer: Ask each question in Table 4. If not spontaneously addressed, ask each probe.]

Table 4.	SD NRS debriefing

| Topic | Question | Probes | Notes |
| --- | --- | --- | --- |
| **Understanding** | In your own words, what does this question mean to you? | *What does “no sleep loss related to itch” mean to you?*  *What does “I cannot sleep at all due to itch” mean to you?* |  |
| **Clarity** | Is there anything about this question that is difficult to understand or respond to?  If not, how could it be made clearer? | *How did you determine your sleep loss related to the symptoms of CPUO?*  *How did you select your response on the scale?* |  |
| **Rewording** | If yes, how would you suggest the question be reworded? |  |  |
| **Recall Period** | What time-period did you think about when you answered the question? | *What time-period were you thinking about when you answered the scale?*  *What does the time frame “last night” mean specifically for you?* |  |
| **Ease** | How easy did you find it to select a response from 0 to 10? Why? | *How easy or difficult was it to select a response on the scale which accurately represented your sleep last night?* |  |

Qualitative exploration of meaningful change and thresholds in SD NRS

Thank you. For the next phase of the interview, I would like to explore with you which changes in the SD NRS would matter to you.

Earlier, you told me your sleep disturbance was [*share score*] last night. Next, I’m going to ask you to think how much a change on this 0-10 scale would be important to you and why.

1. From your score [*share score*] last night what change would be meaningful or important to you in terms of improvement? In other words, what number on the scale would you move to feel that is a noticeable and meaningful improvement in your sleep?

*Insert Number (severity)*

[Interviewer: Say the number participant provided]

2. Can you describe what your sleep looks like at that level? How would your sleep change if you had moved to this level (probes: less frequent or shorter awakenings, increase time of sleep [if so, how much time], reduced time to fall asleep etc.)?

**Probes for Meaningful Change**

a. Would an improvement from [*original* *score reported by the participant*] to [*1-point improvement*] mean something to you?
i. Probe: What could you do differently? Would this change be meaningful or important to you?
b. Would an improvement from [*original* *score reported by the participant*] to [*2-point improvement*] mean something to you?
i. Probe: What could you do differently? Would this change be meaningful or important to you?
c. Would an improvement from [*original* *score reported by the participant*] to [*3-point improvement*] mean something to you?
i. Probe: What could you do differently? Would this change be meaningful or important to you?
d. Would an improvement from [*original* *score reported by the participant*] to [4*-point improvement*] mean something to you?
i. Probe: What could you do differently? Would this change be meaningful or important to you?
3. From your score [*share original score*] last night, what change would be meaningful or important to you in terms of worsening? In other words, what number on the scale would you move to feel that is a noticeable and meaningful worsening in your sleep?

*Insert Number (severity)*

[Interviewer: Say the number participant provided]

4. Can you describe what your sleep looks like at that level? How would your sleep change if you had moved to this level (probes: more frequent or longer awakenings, reduced time of sleep [if so, how much time], increased time to fall asleep etc.)?
5. [Interviewer: If not already covered] Is there a time when you were at the worst point 10? Please describe your experience in terms of sleep disturbance (probes: difficulties to fall asleep, number and duration of awakenings, etc.) at the worst point 10.
6. [Interviewer: If not already covered] How about when you were at the middle point? Please describe your experience in terms of sleep disturbance (probes: difficulties to fall asleep, number and duration of awakenings, etc.) at the middle point (5)?
7. [Interviewer: If not already covered] How about if you were at the lower end of the scale (1-2)? Please describe your experience in terms of sleep disturbance (probes: difficulties to fall asleep, number and duration of awakenings, etc.) at the lower end of the scale (1-2)?

I want you to do an exercise with me. If I asked you to give me ranges for sleep disturbance when it’s not present , mild, moderate and severe.

- What would the not present range be? ______ to ______
- What would the mild range be? ______ to ______
- What would the moderate range be? ______ to ______
- What would the severe range be? ______ to ______

Part 4: Cognitive debriefing of PGIS (15 min)

Cognitive debriefing of the Patient Global Impression of Severity of Pruritus (PGIS)

Thank you. For the next part of the interview, I’d like to focus on the **Patient Global Impression of Severity of Pruritus (PGIS)** Please take a minute to answer the question.

1. What score did you choose?

Please choose the response below that best describes the severity of your pruritus over the past week.

- None
- Mild
- Moderate
- Severe

[Interviewer: Say the answer the participant provided]

2. Can you describe what severity of CPUO looks like at that level?

[Interviewer: Ask each question in Table 4. If not spontaneously addressed, ask each probe.]

Table 5.	PGIS debriefing

| Topic | Question | Probes | Notes |
| --- | --- | --- | --- |
| **Understanding** | In your own words, what does this question mean to you? |  |  |
| **Difference** | Let’s look at the difference between some of these responses. Can you please tell me the difference between… | *Mild and*  *Moderate*  *AND*  *Moderate and severe* |  |
| **Clarity** | Is there anything about this question that is difficult to understand or respond to?  If not, how could it be made clearer? | *How did you determine your impression of severity related to the symptoms of CPUO?*  *How did you select your response?* |  |
| **Rewording** | If yes, how would you suggest the question be reworded? |  |  |
| **Recall Period** | What time-period did you think about when you answered the question? | *What time-period were you thinking about when you answered the question?*  *What does the time frame “past week” mean specifically for you?* |  |
| **Ease** | How easy did you find it to select a response? Why? | *How easy or difficult was it to select a response on the scale which accurately represented your global impression of severity of pruritus over the past week?* |  |

Part 5: Cognitive debriefing of PGIC (15 min)

Cognitive debriefing of the Patient Global Impression of Change of Pruritus (PGIC)

Thank you. For the next part of the interview, I’d like to focus on the **Patient Global Impression of Change of Pruritus (PGIC).** Importantly, this question is asking you to assume or pretend like you are currently participating in a clinical trial. Please take a minute to answer the question.

1. What score did you choose?

Please choose the response below that best describes the overall change of your pruritus since you started taking the study medication:

- Very much better
- Moderately better
- A little better
- No change
- A little worse
- Moderately worse
- Very much worse

[Interviewer: Say the answer the participant provided]

2. Can you describe what change of CPUO looks like at that level?

[Interviewer: Ask each question in Table 4. If not spontaneously addressed, ask each probe.]

Table 6.	PGIC debriefing

| Topic | Question | Probes | Notes |
| --- | --- | --- | --- |
| **Understanding** | In your own words, what does this question mean to you? |  |  |
| **Difference** | Let’s look at the difference between some of these responses. Can you please tell me the difference between… | *Moderately better and A little better* |  |
|  |  | *A little worse and Moderately worse* |  |
| **Clarity** | Is there anything about this question that is difficult to understand or respond to?  If not, how could it be made clearer? | *How did you determine your impression of change related to overall status*  *How did you select your response?* |  |
| **Rewording** | If yes, how would you suggest the question be reworded? |  |  |
| **Recall Period** | What time-period did you think about when you answered the question? | *What time-period were you thinking about when you answered the question?*  *What does the time frame “since you*  *started taking the study medication” mean specifically for you* |  |
| **Ease** | How easy did you find it to select a response? Why? | *How easy or difficult was it to select a response on the scale which accurately represented your change in overall status?* |  |

Part 6: Conclusion (5 min)

Are there any other aspects of your Chronic Pruritus of Unknown Origin, including symptoms and impact, that you would like to discuss? If so, tell me more about that.

Thank you for participating in our study today. Your input has been very helpful.

I will now turn off the recorder.

**Thank you again!**

# Appendix C. Concept elicitation – chronic itch–related signs and symptoms prior to study entry: Interviewers’ probe checklist

| Symptoms | Notes |
| --- | --- |
| Itch |  |
| Lesions/sores |  |
| Skin pain |  |
| Burning |  |
| Stinging |  |
| Tingling |  |
| Hot/cold sensation |  |
| Raw skin |  |
| Dry/rough skin |  |
| White or dark crusts on skin |  |
| Bleeding/scabbing |  |
| Darkening of or purple skin |  |
| Other: __________ |  |

# Appendix D. Concept elicitation – impacts of CPUO prior to study Interviewers’ probe checklist

| Impact description | Notes |
| --- | --- |
| **Day-to-day activities**  Chores/housework/gardening  Daily routine/planning  Getting dressed/what you wear  Self-care/personal hygiene practices |  |
| **Work or school**  Difficulty concentrating |  |
| **Feelings or mood**  Depression  Anxiety  Embarrassment or shame  Sadness  Worry  Low self-esteem  Distress  Difficulty enjoying oneself  Other |  |
| **Social life**  Social activities (e.g., going out to the cinema, bar or for dinner)  Leisure activities/hobbies  Sports (e.g., swimming) |  |
| **Relationships**  Family, romantic or friend relationships  Work/school relationships  Stigma |  |
| **Sleeping**  Length of time needed to fall asleep  Number of awakenings related to chronic itch or associated symptoms  Length of time of awakenings related to chronic itch or associated symptoms  Quality of sleep  Duration of sleep  Waking up rested |  |
| **Other: specify** |  |

Abbreviation: CPUO, chronic pruritus of unknown origin.

# Appendix E. Sociodemographic questionnaire

Please answer the following questions with the best answer.

1. What is your age? _____________
2. What sex were you assigned at birth?

□_1_ Male

□_2_ Female

3. What is your ethnic background?

□_1_ Hispanic or Latino

□_2_ Not Hispanic or Latino

4. What is your racial background (Please check all that apply)?

□_1_ American Indian or Alaska Native

□_2_ Asian

□_3_ Black or African American

□_4_ Native Hawaiian or other Pacific Islander

□_5_ White

□_6_ Other ____________________________

5. What is your current marital status?

□_1_ Single

□_2_ Married

□_3_ Divorced

□_4_ Separated

□_5_ Widowed

□_6_ Other ___________________________

6. What is your current living/domestic situation?

□_1_ Living alone

□_2_ Living with a spouse, partner, family, or friends

□_3_ Other ____________________________

7. How would you describe your employment status? (Please check all that apply)

□_1_ Full-time work

□_2_ Part-time work

□_3_ Homemaker

□_4_ Student

□_5_ Unemployed

□_6_ Retired

□_7_ Disabled

Is your disability related to your chronic itch: □ Yes □ No

□_8_ Other ____________________________

8. What is the highest level of education you have completed? (Please select one answer)

□_1_ Elementary/primary school

□_2_ Secondary/high school

□_3_ Technical or vocational degree

□_4_ Some college/university

□_5_ College /university degree (BA, BS)

□_6_ Postgraduate degree (MA, PhD)

□_7_ Other ____________________________

9. How would you say your general health was within the past month? (Please check one)

□_1_ Very Good

□_2_ Good

□_3_ Fair

□_4_ Poor

10. Please list any other dermatological conditions that you have been diagnosed with by a healthcare provider

□_1_ Acne

□_2_ Hives (Urticaria)

□_3_ Other____________________________
